# Multidisciplinary Clinicians’ Perspectives on Elder Mistreatment: Challenges and Opportunities in Primary Care

**DOI:** 10.1093/geroni/igaf122.4268

**Published:** 2025-12-31

**Authors:** Monique Pappadis, Monique Brown, Karen Schlag, Leila Wood

**Affiliations:** University of Texas Medical Branch at Galveston, Galveston, Texas, United States; University of South Carolina, Columbia, South Carolina, United States; University of Texas Medical Branch at Galveston, Galveston, Texas, United States; University of Texas Health Science Center Houston, Houston, Texas, United States

## Abstract

Elder mistreatment (EM) is an underrecognized public health concern, especially among older adults with mild cognitive impairment or Alzheimer’s Disease and Related Dementias (MCI/ADRD). However, little is known about the perspectives of healthcare professionals on identifying EM in outpatient primary care settings. This qualitative study aimed to explore the perspectives of primary care providers on the identification of EM in older adults with cognitive impairments and cultural considerations in screening. A multidisciplinary sample of 24 outpatient care providers were recruited and interviewed via Zoom. The interview included questions on identification/recognition of EM, cultural considerations in screening, and considerations of addressing EM with patients with MCI/ADRD. Thematic analysis was used to identify recurring themes. Notable themes included: easily detectable visible signs/symptoms, challenges with distinguishing elder mistreatment from common symptoms of dementia, unmet social needs as risk factors, financial strain/dependency on the older adults’ finances, caregivers’ need for respite care to reduce caregiving strain, family neglecting to provide basic needs and cleanliness, and family dynamics and silence during clinical encounters. Cultural considerations included the differences in how individuals define EM, hesitancy for seeking assistance, perceptions of caregiver burden, perceptions of respect towards older adults and the provision of care, and screening in one’s primary language. Primary care clinicians recognize that EM screening and clinician training are needed to address EM in primary care. Building trust between the clinician, older adult and family are essential to identify EM early and address unmet needs of patients with MCI/ADRD and their families.

